# Association between Participation in Counseling and Retention in a Buprenorphine-Assisted Treatment Program for People Experiencing Homelessness with Opioid Use Disorder

**DOI:** 10.3390/ijerph182111072

**Published:** 2021-10-21

**Authors:** Amanda R. W. Berry, Tracy L. Finlayson, Luke M. Mellis, Lianne A. Urada

**Affiliations:** 1 School of Social Work, San Diego State University, San Diego, CA 92182, USA; lurada@sdsu.edu; 2 School of Public Health, San Diego State University, San Diego, CA 92182, USA; tfinlays@sdsu.edu; 3 Saint Vincent de Paul Village, Inc., San Diego, CA 92102, USA; luke.mellis@neighbor.org; 4 Department of Medicine, University of California San Diego, La Jolla, CA 92093, USA

**Keywords:** homelessness, opioid, medication-assisted treatment, buprenorphine

## Abstract

The opioid epidemic is a public health crisis that disproportionately affects our unsheltered neighbors. Because medication-assisted treatment (MAT) is effective for preventing deaths from drug overdose and retention is associated with better health outcomes, there is a clear need for more research on factors impacting retention in care. This retrospective cohort analysis examines the relationship between attendance in counseling and retention on buprenorphine for three or more months for individuals experiencing homelessness being treated at a Federally Qualified Health Center (FQHC) and Public Health Service Act §330(h) Health Care for the Homeless Program grantee in San Diego County, California. The cohort included 306 adults experiencing homelessness who had at least one prescription for buprenorphine and participated in a MAT program between 2017 and 2019. The sample included 64.4% men, almost exclusively white, and 35% lived in a place not meant for human habitation. Of the sample, 97 patients were retained at 3 months and 209 were not. Results from a logistic regression model showed that counseling appointments were positively associated with retention at three months (OR = 1.57, *p* < 0.001). Findings from this study inform future MAT program design components for people experiencing homelessness.

## 1. Introduction

The opioid epidemic in the United States is a public health crisis that disproportionately affects unsheltered populations. Compared to national averages, people experiencing homelessness have higher rates of substance use disorders and higher mortality rates from opioid overdose. According to the 2018 National Survey on Drug Use and Health, 10.3 million people over the age of 12 misused opioids in the past year [1]. Opioid misuse can be fatal. In 2017, overdoses involving opioids accounted for 47,600 deaths in the United States [2]. Unfortunately, these surveys often exclude people with no fixed address, making data on the severity of the problem for unstably housed persons hard to quantify. The 2019 Point in Time Count showed 151,278 individuals in California experiencing homelessness [3]. In a Boston study of people experiencing homelessness, researchers found that drug overdose was the cause of death for a third of adults under the age of 45, and opioids were involved in 81% of the overdose deaths [4].

Declared a national public health emergency in October 2017, the opioid crisis is often viewed through the lens of a medical model of health and purported to be caused by overprescribing and shifting priorities in the medical community to better address pain management [5]. However, the cause of the opioid epidemic goes beyond changes in prescribing practices. If viewed through the lens of social and structural determinants of health, the root causes of the epidemic are revealed in economic disadvantage, limited social capital, and hopelessness [6,7]. When viewing the crisis through this lens, the need for wrap-around services to address the structural environment and housing stability for unstably housed people who are also struggling with opioid use disorder becomes more apparent. The response to upstream social and structural determinants of the crisis has been slower than the micro and mezzo responses in clinical care practices and organizational policy. 

Medication-assisted treatment (MAT), a combination of medication and counseling or behavioral therapies, is considered the standard of care for treating opioid use disorder, yet it is markedly underutilized. This disparity is attributed to a lack of prescriber and provider knowledge, health plan authorization hurdles, workforce shortages, and stigma [8]. Because MAT is effective for preventing deaths from drug overdose and retention in care is associated with better health outcomes, there is a clear need for more research on factors impacting retention, particularly among people who are homeless or at risk of becoming homeless. The National Institute on Drug Abuse notes the effectiveness of medications such as buprenorphine, methadone, and naltrexone for the treatment of opioid use disorders. Despite their effectiveness, access remains a key barrier. Clemans-Cope, Epstein, and Wissoker (2018) found that 48 to 70 percent of people with opioid use disorder (OUD) lack access to these medications [9]. The Centers for Medicare and Medicaid Services (2020) note that only 20% of people with diagnosed OUD receive treatment [10].

The most common medications used in MAT are methadone, buprenorphine, and naltrexone. Buprenorphine is an opioid partial agonist that binds receptors in the brain, but only with partial efficacy. This limits the intoxicating effects compared to full agonists like heroin or methadone [11]. While methadone is dispensed in daily doses by licensed specialty clinics, buprenorphine can be dispensed from a patient’s primary care setting, potentially reducing stigma and improving access to treatment. There are three phases of buprenorphine treatment: induction, stabilization, and maintenance. During induction, a patient must not have taken opioids for 12–24 h and must be experiencing withdrawal when the medication is initially administered. The stabilization phase occurs when the patient is no longer having opioid cravings and is experiencing few side effects. Finally, the maintenance phase is when the patient is on a dose of buprenorphine consistent with their needs; this phase is potentially indefinite for some. 

The integration of MAT into the clinical operations of Federally Qualified Health Centers (FQHCs) aims to address multiple issues in a whole-person, wrap-around care model [12]. Most studies on MAT have been in primary care, hospital, and university settings, which highlights the need for analysis in FQHCs and homeless populations. This study focuses principally on buprenorphine treatment, which is only one medication classified under MAT. The gap in research with low-income populations served by safety net providers such as FQHCs may undercount the barriers and facilitators to retention in care that are associated with social determinants, such as stable housing. 

Housing stability is a social determinant of health that impacts trauma exposure, health care access, and life expectancy. Due to their housing condition, unstably housed people often experience hopelessness and have a clear economic disadvantage, potentially exacerbating their risk of developing opioid use disorder. This demonstrates the need for treatments that target addiction and simultaneously tackle psychological challenges and economic disadvantage. Housing instability is often comorbid with other physical and behavioral health conditions. Opioid misuse at the intersection of health and housing presents programs with an imperative to practice whole-person, patient-centered care that focuses on the strengths and needs of the patient in the context of their environment.

There are unique challenges to caring for homeless individuals and those with unstable housing. Many individuals have limited or unreliable access to telephone and internet services, are often not found in the same location each day, do not have insurance, and may not trust the health care system. Homeless individuals are disproportionately impacted by this epidemic and are nine times more likely to die from an overdose than stably housed individuals [4]. This research analyzes data from the St. Vincent de Paul Village Health Center (VHC), a Health Care for the Homeless FQHC. The Village Health Center serves approximately 3000 patients annually in an integrated care model embedded within a larger homeless services organization, Father Joe’s Villages. They have been establishing trust in the San Diego area since the 1980s and are a known resource for people experiencing homelessness and community organizations seeking to make referrals for clients who are homeless or at risk of homelessness. 

Retention in treatment is a primary outcome in addressing OUD and is associated with reduced drug use, improvements in quality of life and social functioning, and decreases in mortality [13]. The purpose of this retrospective cohort analysis is to examine the relationship between attendance in counseling (individual or group sessions with an alcohol and drug counselor, behavioral health clinician, or psychiatrist) and retention in medication-assisted treatment at three or more months for individuals experiencing homelessness being treated at the VHC. While retention is generally considered to be continuous treatment with gaps of acceptable limits, the operationalization of the measure varies across the literature and ranges from three months to seven years [14]. This study uses the three-month time point to examine how psychosocial supports and polysubstance use impact homeless people’s retention in care. 

## 2. Materials and Methods 

Secondary data were obtained from the VHC. As an FQHC, no individual can be denied care, but all patients seen regularly at VHC must be experiencing homelessness, at risk of becoming homeless, or within 12 months of being homeless as defined under §330(h) of the Public Health Service Act [15]. In 2019, the VHC served 3000 individuals through 17,300 patient visits for primary care, dental care, psychiatry, substance use disorder treatments, and behavioral health services. 

This analysis focused on a cohort of individuals seen in the program’s medication-assisted treatment (MAT) program who received buprenorphine between September 2017 and February 2020. The data were extracted by VHC staff from the health center’s electronic health record, Athena, and combined with individual-level data on patient encounters documented through their Homeless Management Information System (HMIS). Relevant data were extracted from the electronic systems to a de-identified dataset for secondary analysis. The study was approved by the Institutional Review Boards (IRB) at San Diego State University and Father Joe’s Villages. 

The treatment protocol for patients seen in the MAT program at the VHC involves an initial induction visit which includes a urine drug screen, pregnancy test, and liver function tests. Then, the patient receives an intake with a prescribing physician and an evaluation of appropriate detoxification needs, either at the Comprehensive Treatment Center Hub or an out of office induction coupled with a warm-handoff to an Alcohol and Other Drug (AOD) Counselor. Once inducted, patients will meet with the prescribing practitioner to receive weekly prescriptions that were accompanied by a urine drug screen for substance use that would impact treatment and participate in a group counseling session. Potential side effects of medications are proactively discussed with patients and addressed as they occur. All patients have additional access to individual counseling sessions with AOD counselors, licensed behavioral health providers, and visits with a primary care provider to address health needs. VHC policy stipulates that patients in detox who are unable to meet the expectation for weekly follow-up visits must present for a clinic appointment every one to two months to continue buprenorphine treatment. 

There was a total of 308 patients who received a prescription for buprenorphine between 1 January 2017 and 29 February 2020. Moreover, 29 February 2020 was used as the cut-off date for the study due to protocol changes that went into effect in March 2020 with the COVID-19 pandemic. Medications under the umbrella of buprenorphine include buprenorphine-naloxone, buprenorphine HCL, Sublocade^®^, Suboxone^®^, and Zubsolv^®^. Only 20 patients received Sublocade^®^, which is a subcutaneous extended-release injectable; all others are taken as a sublingual film or tablet. Of the 308 patients, 306 had a diagnosis of OUD and were included in the study sample. 

The outcome variable of interest was retention in treatment for three or more months. Because opioid use disorder is a chronic, relapsing disorder, the literature suggests a flexible definition of retention and there is no standardized definition to date [14]. The initial dataset included prescription type and the date prescribed for each patient. A derived variable reflecting months of retention was determined manually in Excel using dates of prescriptions written for each patient. According to the VHC’s prescribing protocols for medication-assisted treatment, patients ideally have prescription refills for buprenorphine-naloxone weekly or every two weeks, and Sublocade refills monthly. SAMHSA TIP #63 notes that frequency of office visits for buprenorphine should “begin daily to weekly then be tailored to the patient’s needs” (Page 61, https://store.samhsa.gov/product/TIP-63-Medications-for-Opioid-Use-Disorder-Full-Document/PEP21-02-01-002, accessed on 30 August 2021). The expected variability in treatment plans informed decisions on how to derive months retained with an acceptable one-month treatment gap for each patient from prescription dates. Patients with three or more months retained were the outcome of interest compared to patients with less than three months retained, the reference category for the dichotomous outcome variable. 

In consultation with VHC staff, multiple prescriptions on the same date were removed and considered duplicates because they likely indicated changes in the pharmacy for pick up rather than independent prescription fills. The first prescription for a patient was considered their induction date and was counted as month one. All subsequent prescriptions were labeled by their order date month with induction referenced as the first month of treatment. Patients were coded for each month of treatment as retained or not retained for that month if they had at least one prescription. Patients with a total of only two prescriptions ever and a gap of more than two months between them were coded as retained for zero months. Gaps in care are common and perfect adherence and compliance is unlikely for this patient population. If they had a prescription in their first month and their third month, they would have counted as retained for two months. Series of months with a prescription were counted for retention over time. Patients were considered discontinued if they had two consecutive months without a prescription. If the patient had a discontinuation and then had a subsequent month with a prescription, this qualified as a “restart.” The longest duration of consecutive months of retention without a restart was counted as the total months retained. The final outcome variable was dichotomized to reflect whether or not patients were retained for three or more months.

Demographic variables included in the original dataset included age, sex, sexual orientation, gender identity, marital status, race, ethnicity, veteran status, income, education, and housing status. Health variables included OUD diagnosis defined as ICD-10 category codes F11-, psychiatric diagnosis for a mood disorder defined as ICD-10 category codes F30-39, psychiatric diagnosis for an anxiety disorder defined as ICD-10 category codes F40-48, buprenorphine prescription type, dosage and dates, urinalysis dates and results, date of encounter for individual and group counseling sessions categorized as behavioral health, alcohol and other drug, or psychiatry, and finally, total number of health care visits at VHC. 

Of the variables provided, sexual orientation, gender identity, marital status, race, veteran status, and education were not included in the analysis due to limited variability or large amounts of missing data. Total visits at VHC were not considered because mapping the visit dates to the treatment period was not possible. The patient’s treatment period was defined as the induction date to 29 February 2020. The variables selected for analysis were categorized by predisposing, enabling, need, and health behavior domains from the Behavioral Model for Vulnerable Populations. Predisposing variables included age, sex, and ethnicity. Housing status and income were considered enabling variables. Need variables included: mood and anxiety disorder diagnoses and other substance use including cannabis, methamphetamine, benzodiazepines, cocaine, and MDMA. Attendance at counseling appointments was a discrete count variable for health behavior. 

Among the 308 patients with a buprenorphine prescription, 2 did not have a diagnosis documented for an opioid use disorder, as defined by the ICD-10 code category F11, and were excluded from analysis, leaving a final analytic sample of 306 patients. Because the VHC’s program is low barrier, the aim is for induction to occur on the same day that the patient presents themselves. However, a patient may present to the clinic, be induced, and then never return for ongoing treatment, which could be due to a transfer to another community provider, stigma, readiness for change, or diversion of medication. Even if patients were considered discontinued, they may have maintained a path toward recovery, but were not captured in the sample data over time. 

The data in this study were analyzed using Statistical Package for the Social Sciences (SPSS) Statistics Version 27 (IBM Corp, Armonk, NY, USA). First, univariate frequencies for each variable were conducted to describe the sample. Then, a bivariate analysis for each independent variable crossed with the dependent variable for retention at three months was used to further describe the data. Covariates included age, sex, ethnicity, sheltered status, income, mood disorder diagnosis, anxiety disorder diagnosis, use of cannabis, methamphetamine, benzodiazepines, cocaine, or MDMA, and counseling encounters. A Pearson’s chi-square test of independence was conducted to explore the bivariate associations between the categorical independent variables and the outcome of interest. For counseling encounters, a count variable, the normality was assessed using the Shapiro–Wilk test, which showed that the number of counseling encounters was not normally distributed (*p* < 0.001). Because the assumption of normality was violated, instead of a two-sample *t*-test, an independent samples Mann–Whitney U Test was used to analyze the difference in counseling encounters between patients retained or not retained at three months. 

Multivariable modeling and analyses were conducted to address the research question: how is participation in counseling sessions associated with retention on buprenorphine at three months? The null hypothesis is that there is no association between the number of attendances in counseling sessions and three or more months’ retention on buprenorphine. With a dichotomous outcome variable (retained or not retained at three months), a binary logistic regression model was appropriate for analysis. The inclusion of covariates was based on the framework developed by Gelberg, Anderson, and Leake (2000), and compared empirically for model fit using a backward selection strategy [16]. While the backward selection strategy indicated the removal of age, mood and anxiety disorders, and methamphetamine use, they were maintained in the model due to established importance in the literature for outcomes for homeless individuals. 

## 3. Results

Table 1 describes the demographics for the total sample (N = 306), and distribution by outcome category. In the total sample of 306, there were 97 (31.7%) retained at three months and 209 (68.3%) not retained at three months. The age split for the sample was slightly favored to under 40 years old (55.6%) compared to over 40 (44.4%). No association was found between retention and age. The sample was predominantly white (91%) and male (64.4%). Among the 306 patients 23.9% were Hispanic or Latino. No association was found between retention and sex, nor retention and ethnicity in the bivariate.

In terms of enabling characteristics, 107 (35%) reported being unsheltered. There was a significant association between being unsheltered and retention at three months, χ2(1, N = 306) = 5.28, *p* = 0.02. Need factors included having a mood disorder diagnosis (24.8%) or an anxiety disorder diagnosis (26.1%). There was a significant association between having a mood disorder diagnosis and retention at three months, χ2(1, N = 306) = 23.12, *p* < 0.001. There was a significant association between having an anxiety disorder diagnosis and retention at three months, χ2(1, N = 306) = 33.30, *p* < 0.001. For polysubstance use, a need factor, 26.1% used benzodiazepines, 16.3% used cocaine, 26.8% used MDMA, 47.4% used cannabis, and 69.0% used methamphetamine. There was a significant association between benzodiazepines, cocaine, MDMA, and cannabis, and retention (*p* < 0.001), but not between methamphetamine and retention. The health behavior of interest in this analysis was participation in counseling, through the variable “counseling encounters”, which had a mean of 3.87 visits across the full sample and a standard deviation of 7.17 visits ranging from 0 to 44 visits. The number of counseling encounters for patients who were retained for three or more months (Median = 7.0) was higher than those for patients not retained (Median = 0.0). Table 2 shows a Mann–Whitney U Test indicating that this difference was statistically significant, U(N_retained_ = 97, N_not retained_ = 209,) = 17,916.5, z = 11.49, *p* < 0.001. 

Table 3 summarizes the regression model results. Among predisposing factors, individuals who identified as Hispanic or Latino were 3.95 times more likely (95% Confidence Interval (CI) = 1.60–9.72, *p* = 0.003) to be retained at three months compared to Non-Hispanic or Latino patients. Unsheltered patients were less likely to be retained at three months (Odds Ratio (OR) = 0.29, CI = 0.11–0.72, *p* = 0.008) compared to sheltered patients. Patients who used cannabis during the treatment time period were 3.52 times more likely to be retained at three months (CI = 1.54–8.04, *p* = 0.003) than those who did not test positive for cannabis. Patients who used methamphetamine during the treatment time period were less likely to be retained at three months (OR = 0.36, CI = 0.137–0.96, *p* = 0.041) than those who did not test positive for methamphetamine. Patients who used MDMA during the treatment time period were 3.65 times more likely to be retained at three months (CI = 1.41–9.44, *p* = 0.008) than those who did not test positive for MDMA. The patient’s health behavior, participation in counseling, was statistically significant. For each additional counseling encounter, a patient was 1.57 times more likely to be retained at three months (CI = 1.35–1.82, *p* < 0.001).

## 4. Discussion

The results of this retrospective cohort study of individuals experiencing homelessness demonstrated that participation in counseling sessions was positively associated with retention on buprenorphine at three or more months. Additional significant associations were observed for ethnicity, housing status, and polysubstance use variables. 

In this study, 32% were retained at three months and only 3% were retained after one year. The three-month results are comparable to other studies. A San Francisco study of people experiencing homelessness showed 27% retention at three months and 18% at 12 months [17]. A New York study of a low-barrier harm reduction program showed 42% retention at three months and 20% at 12 months [18]. Both the San Francisco and New York programs separately reported maintenance on buprenorphine and engagement in care, the latter of which was greater in both cases. Compared to office-based opioid treatment programs, which show more than half of patients retained at one-year, programs serving people experiencing homelessness have much higher rates of disengagement [19]. Monthly retention was defined with flexible parameters that allowed for patients to have some gap in their prescriptions to account for variability in treatment plans, the chronically relapsing nature of substance use disorders, and the vulnerability of the patient population. Potential interruptions in treatment could have been due to justice involvement, hospitalization, or transition to methadone treatment. According to VHC staff, there are other possible explanations for a patient to have only received one prescription. Potential reasons include that: they intend to sell the medication or divert it for use by someone else, they plan to use the prescription as an emergency backup drug when other opioids are not available, the program requirements are too restrictive, stigma, they view buprenorphine as a detox rather than long-term therapy, and lastly, substance use is a component of their social support network that they rely on for safety. Side effects of buprenorphine could also be a reason; however, VHC staff reported that side effects were not a barrier for the patient population seen in the clinic. 

Hispanic or Latino patients were more likely to be retained in this sample, which contrasts with the existing literature for office-based opioid treatment retention at one year [19]. San Diego is a border community and most VHC providers are bilingual, which could facilitate better outcomes for those patients. The study sample was racially homogeneous (91% white, 5% black) and outreach and engagement in health-care seeking could be improved, for black San Diegans in particular. Black people make up nearly 5% of the population but 21% of the unsheltered homeless population and 30% of the sheltered homeless population in San Diego (“WeAllCount”, 2020). Black people use opioids at lower rates compared to white people, but are increasingly dying from overdoses at higher rates, reinforcing the need to address this disparity. 

In addition to racial disparities in health, social inequities in the lived environment for people experiencing homelessness can exacerbate the risk of substance use. Homelessness is not a universal experience; some individuals reside in shelters, permanent supportive housing, transitional units, couch surf, or double up with others. People experiencing unsheltered homelessness (sleeping outside, in a car, or other places not meant for human habitation for instance) are often homeless for longer and have poorer health and employment outcomes [20]. Shelter is often accompanied by locked boxes or doors to keep belongings safe and prevent loss or theft of medications. Unsheltered patients in this study may have been less likely to be retained due to issues with medication safety and storage. Alford et al. (2007) showed that patients who were homeless had similar outcomes to housed patients; however, this study excluded patients with polysubstance use more than the occasional use and expected daily presentation to the clinic for dosing, which would likely self-select for patients more likely to be retained [21]. 

Polysubstance use is common among those who use nonmedical opioids and has been shown to increase morbidity and mortality among the population [22]. Positive urine tests for cannabis, methamphetamine, and MDMA were significant in this study. Among patients experiencing homelessness seen in primary care facilities, lifetime use of cannabis and cocaine were the most common substances used [23]. Results from this analysis showed that cannabis use was positively associated with retention on buprenorphine, which is similar to existing reports that suggested 21% greater odds of retention with daily use [24]. California legalized marijuana in 2016, making it more accessible and acceptable, and it carries less risk of interacting with law enforcement compared to other drugs. States with legal medical marijuana policies are associated with reduced opioid overdose mortality rates, yet simultaneously face misrepresentation of cannabis as a standalone treatment for OUD [25,26]. Studies on the use of endocannabinoids to facilitate detoxification and addiction management support the results found in this analysis for increased retention with simultaneous use [27]. Cannabis appears to have potential benefits to improve retention, either as a biological facilitator, a harm reduction strategy, or a self-regulation practice that may potentially reduce other psychiatric symptoms. 

Methamphetamine use is a rising concern across the nation, with some claiming “twin epidemics’’ between methamphetamine and opioid use [28,29]. Patients in this study who used methamphetamine, a stimulant, were less likely to be retained in treatment, which is consistent with findings from Tsui et al. (2020) that showed patients who used methamphetamine were twice as likely to drop out of buprenorphine treatment for opioid use [30]. Methamphetamine use is also correlated with opioid overdose among people who inject drugs [31,32]. It is common for people to combine methamphetamine and opioids to enhance a high, balance or compensate for undesired effects, and some even incorrectly presume that a stimulant can reduce the risk of overdose [22,31]. Research has shown that, for patients who use methamphetamine and remain retained on buprenorphine, their methamphetamine use declines over time [30]. Because the current best practice treatments for methamphetamine use are behavioral in nature, future research may consider investigating whether there are spillover effects from counseling received during MAT treatment that improve outcomes for recovery from other substance use disorders. 

MDMA (ecstasy) use during the treatment period was positively associated with retention. This study contributes to the limited evidence about the use of MDMA among people experiencing homelessness. Among youth in Vancouver, Canada, regular MDMA use reduced intravenous drug use (Gaddis et al., 2018) [33]. Future studies should collect and analyze urine toxicology screens to determine whether MDMA was used alone or in conjunction with other drugs. Additional research is needed on the impact of substance use during buprenorphine treatment based on the propensity for polysubstance use both intentionally and unintentionally among people who use drugs. Because patients who take buprenorphine may not completely abstain from opioid use, and urine drug screen results are not available for all patients at all time points, opioid use was not included in this analysis. Still, about 25% of those retained at three months never had a positive urine screen for opioids during their treatment period. Future research should explore this group’s behavior to determine associations between MAT retention and abstinence in the recovery process. 

Counseling encounters in this study were significant in predicting retention, which may be specific to this vulnerable population where psychosocial support and group interactions may confer additional protective factors to the community. Additional research on the type and intensity of the counseling or therapy would be informative, as the literature is mixed regarding the benefits of psychosocial treatment for retention on buprenorphine. Some studies of office-based buprenorphine treatment showed no relationship to engagement in counseling [34]. However, there is significant evidence that cognitive behavioral therapy and contingency management are effective in methadone treatment programs [35]. If psychosocial interventions show little benefit to increasing retention on buprenorphine, and are required for program participation, they may be considered a barrier to treatment. Due to the rapid, widespread implementation of telehealth during the COVID-19 pandemic, future research should explore the impact of virtual counseling visits on reducing barriers to care and retention outcomes. 

Much of the existing literature analyzes MAT programs with restrictive barriers to entry. Some exclude patients who lack housing stability, have co-occurring psychiatric diagnoses, polysubstance use, or have used heroin >4 days in the past month [21,36]. In contrast, the VHC is a low-barrier program that provides flexibility in treatment plans to address substance use goals in the context of housing instability. Because a standardized definition of retention does not exist, this study uses a flexible definition to mirror the program design and account for the vulnerable nature of the population. If a stricter definition of retention were used to match the NQF definition for patients on buprenorphine, one would expect the number of patients who were considered retained to drop precipitously. Moreover, if more restrictive inclusion criteria were a precondition for program entry, one would expect the retention rates to be higher. 

The use of retention as a health outcome may be considered a limitation. The WHO (2009) recommends that unless there is an absence of other outcome measures, retention should be considered an exposure to the intervention rather than a health outcome. In this study, it is important to consider the nuance of this definition. If someone is retained, it is a relative proxy for recovery health outcomes, but individuals may still continue to use opioids while retained in the treatment program. While retention may not be considered a health outcome by some, the benefits of buprenorphine persistence in reducing mortality counterbalance the concerns. Three-month retention is also a short period of time, and examining retention over longer study periods is warranted. Another limitation of retrospective cohort studies using secondary data is reliance on the availability and accuracy of available records. The medical record had gaps and substantial missing data for education and employment that, if complete, would clarify additional social determinants for retention. Urine screens were not available at each prescription time point so could not be matched over time to confirm adherence to the medication or frequency of polysubstance use, which may skew the impact of covariates on retention. There still may be confounding in the association of these variables. Despite the limitations, this study adds important insight to the existing literature regarding the benefits of counseling as an essential intervention associated with persistence in treatment of OUD among people experiencing homelessness PEH. 

## 5. Conclusions

Recovery from substance use is a growing problem, and findings from this study may help inform future MAT programs to promote retention. The positive association found between counseling and retention on buprenorphine highlights the importance of psychosocial supports for people experiencing homelessness engaged in recovery programs. Use of cannabis and MDMA was positively associated with retention, whereas methamphetamine was negatively associated. 

## Figures and Tables

**Table 1:** Descriptive statistics and bivariate analysis.

	Full Sample (N = 306)	Not Retained 3 Months (n = 209)	Retained 3 Months (n = 97)	Pearson Chi-Square
	*n (%)*	*n* (%)	*n* (%)	
Predisposing				
Age 20–39 (ref)	170 (55.6%)	124 (59.3%)	46 (47.4%)	
Age 40–75	136 (44.4%)	85 (40.7%)	51 (52.6%)	3.80
Male (ref)	197 (64.4%)	132 (63.2%)	65 (67.0%)	
Female	109 (35.6%)	77 (36.8%)	32 (33.0%)	0.43
Not Hispanic or Latino (ref)	233 (76.1%)	160 (76.6%)	73 (75.3%)	
Hispanic or Latino	73 (23.9%)	49 (23.4%)	24 (24.7%)	0.10
Enabling				
Sheltered (ref)	199 (65.0%)	127 (60.8%)	72 (74.2%)	
Unsheltered	107 (35.0%)	82 (39.2%)	25 (25.8%)	5.28
No income (ref)	208 (68.0%)	149 (71.3%)	59 (60.8%)	
Any income	98 (32.0%)	60 (28.7%)	38 (39.2%)	3.334
Need				
No mood disorder (ref)	230 (75.2%)	174 (83.3%)	56 (57.7%)	
Mood disorder	76 (24.8%)	35 (16.7%)	41 (42.3%)	23.12 ***
No anxiety disorder (ref)	226 (73.9%)	175 (83.7%)	51 (52.6%)	
Anxiety disorder	80 (26.1%)	34 (16.3%)	46 (47.4%)	33.30 ***
No benzodiazepines (ref)	226 (73.9%)	170 (81.3%)	56 (57.7%)	
Benzodiazepines	80 (26.1%)	39 (18.7%)	41 (42.3%)	19.12 ***
No cocaine (ref)	256 (83.7%)	188 (90.0%)	68 (70.1%)	
Cocaine	50 (16.3%)	21 (10.0%)	29 (29.9%)	19.10 ***
No MDMA (ref)	224 (73.2%)	166 (79.4%)	58 (59.8%)	
MDMA	82 (26.8%)	43 (20.6%)	39 (40.2%)	13.02 ***
No cannabis (ref)	161 (52.6%)	133 (63.6%)	28 (28.9%)	
Cannabis	145 (47.4%)	76 (36.4%)	69 (71.1%)	32.13 ***
No methamphetamine (ref)	95 (31.0%)	69 (33.0%)	26 (26.8%)	
Methamphetamine	211 (69.0%)	140 (67.0%)	71 (73.2%)	1.19

*** *p* < 0.001.

**Table 2:** Mann–Whitney U Test for counseling encounters.

Measure	Not Retained 3 Months		Retained 3 Months		*z*
	*M*	*SD*	*M*	*SD*	
Counseling encounters	0.92	1.89	10.22	9.80	11.49 ***

*** *p* < 0.001.

**Table 3:** Logistic regression model results (N = 306).

Variable	OR (LL–UL)
Predisposing	
Age	0.58 (0.26–1.30)
Sex	1.79 (0.81–3.97)
Ethnicity	3.95 ** (1.60–9.72)
Enabling	
Shelter status	0.29 ** (0.11–0.72)
Income	1.60 (0.696–3.66)
Need	
Mood disorder diagnosis	1.30 (0.52–3.27)
Anxiety disorder diagnosis	1.26 (0.50–3.19)
Cannabis	3.52 ** (1.54–8.04)
Methamphetamine	0.36 * (0.14–0.96)
Benzodiazepines	1.82 (0.759–4.36)
Cocaine	1.41 (0.45–4.40)
MDMA	3.65 ** (1.41–9.44)
Health Behaviors	
Counseling encounters	1.57 *** (1.35–1.82)

*Note.* Total *N* = 306. *** *p* < 0.001, ** *p* < 0.01, * *p* < 0.05.

## Data Availability

Restrictions apply to the availability of these data. Data was obtained from Village Health Center and are available from Luke Mellis (luke.mellis@neighbor.org) with the permission of Village Health Center.
